# Threat and Control of *tet*(X)-Mediated Tigecycline-Resistant *Acinetobacter* sp. Bacteria

**DOI:** 10.3390/foods14193374

**Published:** 2025-09-29

**Authors:** Chong Chen, Taotao Wu, Jing Liu, Jie Gao

**Affiliations:** 1 Joint International Research Laboratory of Agriculture and Agri-Product Safety, Institutes of Agricultural Science and Technology Development, Yangzhou University, Yangzhou 225009, China; 2 College of Bioscience and Biotechnology, Yangzhou University, Yangzhou 225009, China

**Keywords:** *Acinetobacter* spp., food-producing animals, tigecycline resistance, *tet*(X), rapid detection, treatment options

## Abstract

Tigecycline is regarded as one of the last-resort antibiotics against multidrug-resistant (MDR) *Acinetobacter* sp. bacteria. Recently, the tigecycline-resistant *Acinetobacter* sp. isolates mediated by *tet*(X) genes have emerged as a class of global pathogens for humans and food-producing animals. However, the genetic diversities and treatment options were not systematically discussed in the era of One Health. In this review, we provide a detailed illustration of the evolution route, distribution characteristics, horizontal transmission, and rapid detection of *tet*(X) genes in diverse *Acinetobacter* species. We also detail the application of chemical drugs, plant extracts, phages, antimicrobial peptides (AMPs), and CRISPR-Cas technologies for controlling *tet*(X)-positive *Acinetobacter* sp. pathogens. Despite excellent activities, the antibacterial spectrum and application safety need further evaluation and resolution. It is noted that deep learning is a promising approach to identify more potent antimicrobial compounds.

## 1. Introduction

The genus *Acinetobacter* is a complicated group of Gram-negative bacteria from multiple sources, such as human, animal, meat, vegetable, fruit, milk, soil, water, activated sludge, and sewage [1,2,3,4,5]. To date, 87 non-duplicate *Acinetobacter* species have been validly reported (https://www.bacterio.net/genus/acinetobacter, accessed on 6 September 2025). Some of them are well-known opportunistic pathogens for humans and animals, and the most common species is *Acinetobacter baumannii*, followed by *Acinetobacter calcoaceticus*, *Acinetobacter lwoffii*, *Acinetobacter pittii*, and *Acinetobacter junii* [6,7,8]. It is estimated that the global incidence of *Acinetobacter* infections reaches one million cases per year, including ventilator-associated pneumonia, bloodstream infections, and surgical site infections, of which the highest density of infections occurs in intensive care units [6,9]. By contrast, the incidence rate in China and United Arab Emirates is much higher than those reported in the United States and Europe [10,11,12,13]. In addition, the percentage of bloodstream infections in India, Saudi Arabia, and South Africa ranges from 12.4% to 21.3%, with the mortality exceeding 60% [10,14,15,16]. Worrisomely, they are capable of acquiring or up-regulating genetic determinants to develop multidrug resistance, especially the reported resistance to carbapenem and colistin immediately after their clinical application [11,17,18,19]. In 2019, there were 50,000–100,000 deaths caused by carbapenem-resistant *A. baumannii* (CRAb) around the world [20]. Thereafter, CRAb was classified into the critical group by World Health Organization Bacterial Priority Pathogens List in 2024 [21]. The rapid dissemination of MDR *Acinetobacter* sp. pathogens renders tigecycline as one of the last options for their clinical infections.

Tigecycline was first approved by the United States Food and Drug Administration (USFDA) in 2005 and imported into Europe in 2006 as well as China in 2011, which belongs to the third-generation tetracycline [22]. It exhibits a broader-spectrum antibacterial activity than the first- and second-generation tetracyclines by inhibiting bacterial protein synthesis [23]. Unfortunately, the minimum inhibitory concentration (MIC) of tigecycline against *A. baumannii* in Asia was increased when compared with those of Europe and North America [24], of which tigecycline resistance was sporadically detected in 1.7% of clinical *Acinetobacter* sp. strains in China (https://www.chinets.com/Data/AntibioticDrugFast, accessed on 6 September 2025). The main mechanisms contain efflux pumps [e.g., adeABC and *tet*(Y)], altered outer membrane permeability (e.g., *plsC*), altered tigecycline targets of action (e.g., *rpsJ*), tigecycline-inactivating enzymes [e.g., tet(X)], and DNA repair pathways (e.g., *recA*) [25,26,27,28]. Since 2019, the tigecycline resistance mediated by *tet*(X3), *tet*(X4), *tet*(X5), *tet*(X6), *tet*(X7), and *tet*(X15) variants has been reported in global *Acinetobacter* species, especially *bla*_NDM-1_-positive strains in food-producing animals [22,29,30,31,32,33,34]. Nevertheless, genetic diversities and how to prevent the threat of *tet*(X)-positive *Acinetobacter* spp. remain poorly understood. Herein, we intend to explore their *tet*(X) evolution, epidemiology, horizontal transmission, detection, and treatment options.

## 2. Evolution of *tet*(X)-Mediated Tigecycline Resistance

### 2.1. Emergence of tet(X) Genes

**Figure 1:**
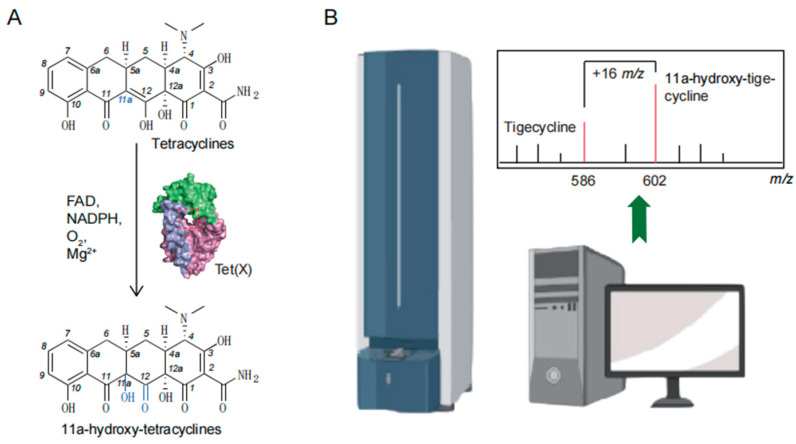
Mechanism of Tet(X) degrading tetracyclines. (**A**) Hydroxylation of tetracyclines by Tet(X). (**B**) Mass spectrometry of tigecycline and its degradation product 11a-hydroxy-tigecycline.

The *tet*(X) gene was first described as *Tc^r^ on an R plasmid and formally named on two transposons Tn*4351* and Tn*4400* in obligate anaerobe *Bacteroides fragilis* [35,36]. It encodes a flavin-dependent monooxygenase Tet(X), which can degrade tetracycline antibiotics by hydroxylation at C11a and requires flavin adenine dinucleotide (FAD), reduced nicotinamide adenine dinucleotide phosphate (NADPH), molecular oxygen, and Mg^2+^ for activity (Figure 1) [37]. Tigecycline was also identified as one of the reaction substrates but failed to reach the USFDA tigecycline resistance breakpoint [30,38]. Then, *tet*(X1) and *tet*(X2) were reported on a transposon CTnDOT of obligate anaerobe *Bacteroides thetaiotaomicron* [39]. The Tet(X1) protein encoded by *tet*(X1) loses its degradation activity due to the lack of 29 amino acids at the N-terminal domain, while the Tet(X2) protein encoded by *tet*(X2) has a similar degradation activity to the first-reported Tet(X) protein [37]. In recent years, there has been an increasing number of novel *tet*(X) variants in the genus *Acinetobacter*, such as *tet*(X3), *tet*(X4), *tet*(X5), *tet*(X6), *tet*(X7), and *tet*(X15), which confer high-level cross-resistance to tigecycline, eravacycline and omadacycline [29,30,31,34,40].

### 2.2. Structural Insights into Tet(X) Proteins

Phylogenetically, *tet*(X) homologs may have originated from chromosomal monooxygenase genes in *Weeksellaceae* (formerly *Flavobacteriaceae*) bacteria, such as *Weeksella* spp., *Chryseobacterium* spp., *Elizabethkingia* spp., *Riemerella* spp., and *Empedobacter* spp. [30,41,42]. All Tet(X) proteins share an overall analogous architecture consisting of the substrate-binding domain, FAD-binding domain, and C-terminal α-helix [30]. The amino acid substitutions at L282, V329, A339, D340, V350, and K351 of Tet(X2) significantly enhance the enzymic degradation activity [40,43]. These substitutions do not directly participate in the binding of tetracyclines and FAD but alter the conformational dynamics of Tet(X) variants through interactions with adjacent amino acid residues [40]. With extensive usage of tetracyclines, the rapid emergence of *tet*(X) variants possibly promote the adaptation of *Acinetobacter* species under antibiotic selection pressure [44]. More researchers are needed to develop novel antimicrobial compounds targeting the six sites for preventing *tet*(X)-positive *Acinetobacter* sp. pathogens.

## 3. Global Epidemiology of *tet*(X)-Positive *Acinetobacter* sp. Strains

### 3.1. Classification of tet(X) Genes

**Figure 2:**
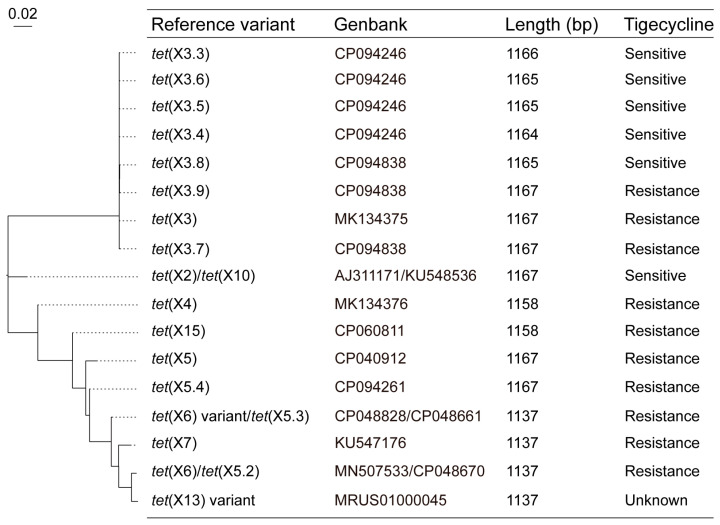
Maximum-likelihood tree of the *tet*(X) variants in *Acinetobacter* spp. GenBank accession number, nucleotide sequence length, and tigecycline susceptibility are also provided.

In total, there are 17 non-redundant *tet*(X) variants reported in *Acinetobacter* app. in eight countries [30,34,45]. These genes include *tet*(X2), *tet*(X3), *tet*(X3.3)-*tet*(X3.9), *tet*(X4), *tet*(X5), *tet*(X5.4), *tet*(X6), *tet*(X6) variant, *tet*(X7), *tet*(X13) variant, and *tet*(X15), with lengths ranging from 1137 bp to 1167 bp (Figure 2). Apparently, three pairs of genes are named repeatedly among them. By multiple sequence alignment of complete nucleotide sequences, *tet*(X2) (AJ311171) and *tet*(X10) (KU548536) share a 100% nucleotide identity; *tet*(X6) (MN507533) and *tet*(X5.2) (CP048670) share a 100% nucleotide identity; and one *tet*(X6) variant (CP048828) and *tet*(X5.3) (CP048661) share a 100% nucleotide identity (Figure 2).

### 3.2. China

**Figure 3:**
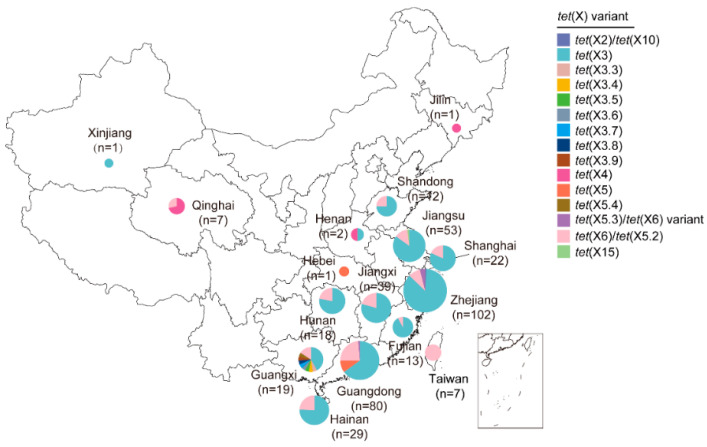
Spread of *tet*(X)-positive *Acinetobacter* spp. in China. The map is mainly generated based on our previous study [30].

**Figure 4:**
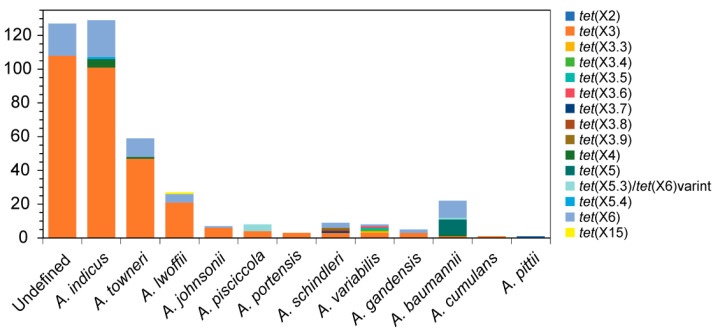
Distribution of *tet*(X) variants in different *Acinetobacter* species in China. The data are mainly collected from our previous study [30].

Statistically, the *tet*(X)-positive *Acinetobacter* sp. strains have been reported in sixteen provinces in China, of which Zhejiang, Guangdong, and Jiangsu provinces covered the majority (>50 isolates per province; Figure 3). By bacterial taxonomy, these strains were widely distributed in 12 different *Acinetobacter* species except the undefined ones (Figure 4). Details are as follows.

#### 3.2.1. Predominate Groups of *tet*(X3) and *tet*(X6)

Since the first report of *tet*(X3) in *A. baumannii*, it has been detected across at least 11 *Acinetobacter* species in 12 provinces of China (Figure 3 and Figure 4) [29,30,46]. Whole genome sequencing (WGS) indicated that *tet*(X3) was the major subtype in *Acinetobacter* spp. (Figure 4), most of which were *Acinetobacter indicus* isolates from animals and neighboring environments [30]. Sporadically, two *Acinetobacter gandensis* strains and one novel *Acinetobacer* species carrying *tet*(X3) were detected in vegetables in Jiangsu and Zhejiang provinces [30]. It is noted that the *tet*(X3) and *bla*_NDM-1_ genes were simultaneously detected in *A. indicus*, *Acinetobacter schindleri*, and *A. lwoffii* [29,30,33]. One study also identified seven novel *tet*(X3) variants [namely *tet*(X3.3)–*tet*(X3.9)] from pig-derived *Acinetobacter variabilis* and chicken-derived *A. schindleri* in Guangxi province, among which only *tet*(X3.7) and *tet*(X3.9) were capable of encoding a functional protein [47]. The *tet*(X6) gene [namely *tet*(X5.2)] was first reported in pig-derived *Proteus* genomospecies 6 in Henan province [48]. To date, it has been detected among at least eight *Acinetobacter* species in 12 provinces in China (Figure 3 and Figure 4), especially with *tet*(X3) in vegetable-derived *A. gandensis* [22,30,49,50,51]. A novel variant of *tet*(X6) [namely *tet*(X5.3)] was reported in *Acinetobacter piscicola* and *bla*_NDM-1_-positive *A. baumannii* from chicken, pig, and soil in Guangdong and Zhejiang provinces [30,52].

#### 3.2.2. Sporadic Groups of *tet*(X2), *tet*(X4), *tet*(X5), and *tet*(X15)

Since the discovery of *tet*(X2) [namely *tet*(X10)] in *B. thetaiotaomicron*, only one human strain of *A. pittii* carrying chromosome-borne *tet*(X2) was isolated in Zhejiang province [39,53]. Since discovering *tet*(X4) in *Escherichia coli* in 2019, a *tet*(X4)-positive *A. baumannii* strain was isolated from inpatients in Jilin province, followed by five *tet*(X4)-positive *A. indicus* strains from migratory birds in Qinghai province [29,30]. An *Acinetobacter towneri* strain carrying chromosome-borne *tet*(X4) was also detected in pig-derived samples in Henan province [54]. The *tet*(X5) gene was first reported in a human-derived *A. baumannii* strain in Hebei province [55]. Subsequently, it was isolated in five duck- and three chicken-derived *A. baumannii* strains in Guangdong province [22]. A novel variant, *tet*(X5.4), was isolated in a pig-derived *A. indicus* strain in Guangxi province [49]. For *tet*(X15), only one chicken strain of *A. variabilis* carrying chromosome-borne *tet*(X15) was discovered in Jiangsu province [31].

### 3.3. Other Countries

The detection of *tet*(X)-positive *Acinetobacter* spp. is relatively rare in seven other countries. Briefly, one tigecycline- and carbapenem-resistant *A. towneri* isolate co-harboring plasmid-mediated *tet*(X7) and *bla*_NDM-1_ was reported in hospital wastewater in Philippines [34]. Based on a source tracking study of global metagenomic data, *tet*(X3), *tet*(X6), and one *tet*(X13) variant were detected in 12 *Acinetobacter* sp. strains of human, pig, and cattle origin from Thailand (*n* = 3), Germany (*n* = 3), Ireland (*n* = 3), Pakistan (*n* = 1), Peru (*n* = 1), and Cote d’Ivoire (*n* = 1), but the degradation activity, genetic environment, and *Acinetobacter* subspecies of the *tet*(X13) variant need further identification [45]. It is noted that the limited epidemiological studies may underestimate the prevalence and transmission risk of *Acinetobacter* spp. in various countries, which could be optimized by WGS techniques.

## 4. Horizontal Transmission of *tet*(X) Genes

### 4.1. Mobilizable Plasmids

So far, the plasmid-mediated *tet*(X3), *tet*(X3.7), *tet*(X3.8), *tet*(X3.9), *tet*(X5), *tet*(X5.3), *tet*(X5.4), *tet*(X6), and *tet*(X7) genes have been reported in diverse *Acinetobacter* species [30,34,47,49,55]. According to multiple sequence alignment of plasmid replicons, these *Acinetobacter* plasmids harboring *tet*(X) genes were mainly classified into six categories, including GR26, GR31, GR41, GR59, GR60, and GR61 [56]. In vitro conjugation studies showed that the plasmid-mediated horizontal transfer event of *tet*(X) genes rarely occurred. Only a few strains can be transferred into the recipient *Acinetobacter baylyi* and *A. baumannii* strains, with an efficiency of about 10^−10^–10^−6^, and imposed significant fitness costs on bacterial growth rates [29,30,47,49,56]. Our previous study confirmed the *tet*(X)-carrying *Acinetobacter* plasmids were complex and only 21.7% of them carried conjugation transfer genes [56]. Consequently, the impaired mobility of plasmid-mediated *tet*(X) genes may be associated with the absence of plasmid conjugation transfer elements, and the specific mechanism warrants more exploration.

### 4.2. ISCR2-Mediated Transposons

IS*CR2*, an insertion sequence (IS) related to the IS*91* family, is considered atypical because it lacks conventional terminal inverted repeats and does not generate target site duplications upon insertion [57]. It is hypothesized to mediate the horizontal spread of antibiotic resistance genes (e.g., *floR* and *sul2*) through a ‘rolling circle’ replication process initiated at *ori*IS and terminated at non-specific *ter*IS [57]. Except for IS*Aba1*-mediated *tet*(X2), IS*26*-mediated *tet*(X7), and IS*Aba1*-mediated *tet*(X15), *tet*(X3), *tet*(X4), *tet*(X5), *tet*(X6), and their variants are also closely related to IS*CR2* in *Acinetobacter* species, with a transposition efficiency of about 10^−8^–10^−6^ into the recipient *A. baylyi* and *E. coli* strains [22,30,31,34,53]. Namely, the typical genetic environment of *tet*(X3) is IS*CR2*-*tpnF*-*tet*(X3)-hp-hp-IS*CR2*, and the upstream IS*CR2* is often truncated by IS*26* and IS*Aba14* [30]; the *tet*(X4) gene has a highly similar genetic environment, namely IS*CR2*-*catD*-*tet*(X4)-IS*CR2*, across *Acinetobacter* species, *E. coli*, *Klebsiella pneumoniae*, and other *Enterobacteriaceae* bacteria [30]; the genetic environment of *tet*(X5) is IS*CR2*-*tpnF*-*tet*(X5)-hp-hp-IS*CR2* [30]; the *tet*(X6) gene has a similar genetic environment between *Acinetobacter* spp. and *Proteus* spp., and its basic structure is IS*CR2*-hp-*tet*(X6)-hp-IS*CR2* [22]. Therefore, IS*CR2* can serve as a potential knockout target to inhibit the horizontal spread of *tet*(X) genes across different bacterial species.

## 5. Rapid Detection Methods of *tet*(X) Variants

With the expanding family of *tet*(X) variants in *Acinetobacter* spp., there is an urgent demand for rapid detection methods to monitor and prevent *tet*(X)-mediated cross-resistance to tetracyclines. Firstly, the specific primers of *tet*(X2), *tet*(X3), *tet*(X4), and *tet*(X5) were successfully designed, and multiplex PCR and multiplex Real-Time PCR assays were developed for screening the *tet*(X) genes in animal, clinical, and environmental samples [58,59]. Based on visual orange to green (OTG) dye, a loop-mediated isothermal amplification assay was applied to simultaneously detect *tet*(X2), *tet*(X3), *tet*(X4), and *tet*(X5), which was significantly more sensitive than usual PCR [60]. Coupled with pH-sensitive bromocresol purple, a rapid detection method was also established based on the degradation of eravacycline by Tet(X3)- or Tet(X4)-producing strains, which resulted in reduced eravacycline activity against an acid-producing thermophile *Bacillus stearothermophilus* indicator strain [61]. An one-tube RPA-CRISPR-Cas12b-based detection system was also developed for specific detection of *tet*(X4) within 40 min [62]. In addition, MALDI^Tet(X)^ and MALDI^Tet(X)-plus^ tests of tigecycline and oxytetracycline were conducted using matrix-assisted laser desorption ionization–time of flight mass spectrometry (MALDI-TOF MS) for rapid detection of Tet(X)-producing Gram-negative bacteria such as *Acinetobacter* sp. strains [63,64]. The evolution of novel *tet*(X) variants and low gene abundance necessitate continuous method updates to maintain detection coverage and accuracy, especially in the field of food-related microbiological detection.

## 6. Treatment Options

### 6.1. Chemical Drugs

There has been no novel antibiotic chemical class with activity against *A. baumannii* that reached patients in over 50 years [65]. In 2024, a novel antibiotic class, tethered macrocyclic peptide (MCP), was reported for the effective treatment of tigecycline-resistant *A. baumannii* infections [65]. A clinical candidate, zosurabalpin (RG6006), was derived from the MCP class and significantly inhibited highly antibiotic-resistant *A. baumannii* isolates in vitro and in vivo [65]. A 90% MIC indicated zosurabalpin (1 mg/L) was much lower than those of tigecycline (8 mg/L), colistin (>16 mg/L), and meropenem (>16 mg/L) [65]. Molecular mechanisms revealed it blocked the transport of bacterial lipopolysaccharide from the inner membrane to its destination on the outer membrane by inhibition of the LptB_2_FGC complex in *Acinetobacter* sp. strains [65,66]. As inhibitors of the membrane lipooligosaccharide transporter MsbA, cerastecins were significantly bactericidal in vitro and in murine models of bloodstream or pulmonary *A. baumannii* infection [67]. Four USFDA-approved drugs, including ZINC000003801919, DB01203, DB11217 and ZINC0000000056652, were identified as efficient inhibitors to combat tigecycline-resistant *A. baumannii* by targeting the BaeR protein [68]. Doxycycline showed a synergistic effect with benzydamine on *tet*(X6)-mediated tigecycline-resistant *A. baumannii* [69]. Toward extended library synthesis of enzymic inhibitors, anhydrotetracycline and semisynthetic analogues were confirmed as competitive inhibitors of Tet(X) enzymes that rescued the activity of tetracyclines against tigecycline-resistant *Acinetobacter* species [70,71,72].

**Figure 5:**
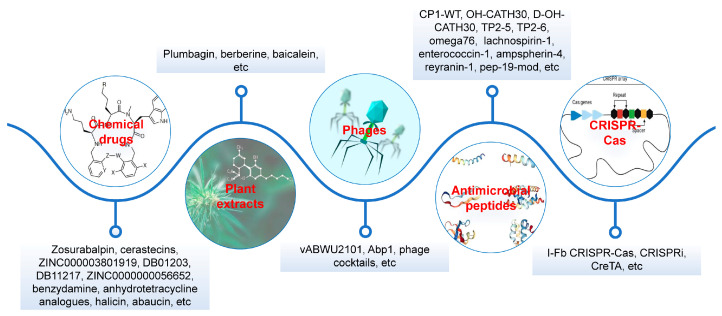
Promising treatment options against *tet*(X)-positive *Acinetobacter* sp. strains.

Deep learning approaches increase the probability of discovering novel chemical drugs (Figure 5). As early as 2020, a broad-spectrum antibiotic, halicin, was discovered through deep learning [73]. The MIC of halicin against clinical MDR *A. baumannii* 288 was 1 mg/L and the bacterial load in a halicin-treated murine wound model was significantly decreased than that of the vehicle control group [73]. A narrow-spectrum antibiotic against *A. baumannii*, abaucin, was also screened by deep learning and could overcome intrinsic and acquired resistance mechanisms in clinical isolates [74]. It perturbed lipoprotein trafficking via LolE, which is a functionally conserved protein that contributes to shuttling lipoproteins from the inner membrane to the outer membrane [74]. The bacterial load and inflammation in the *A. baumannii*-infected mice treated with abaucin were significantly less than those of the vehicle control group [74]. These studies highlight the utility of deep learning approaches to expand our antibiotic arsenal through the discovery of structurally distinct antibacterial molecules.

### 6.2. Plant Extracts

Plant extracts are a class of natural antimicrobials present in various plants (Figure 5). As a naphthoquinone compound, plumbagin was confirmed as a broad-spectrum inhibitor of Tet(X) enzymes by binding to their catalytic pockets and showed a synergistic bactericidal effect with tetracyclines on *tet*(X3)- or *tet*(X4)-mediated tigecycline-resistant strains [75]. Berberine hydrochloride was one of the most common forms of Berberine in Huanglian, and both Berberine hydrochloride and Berberine can restore the antibiotic susceptibility of MDR *A. baumannii* to tigecycline, meropenem, ciprofloxacin, and sulbactam [76,77]. Baicalein extracted from *Scutellaria baicalensis* also exhibited a synergistic activity with tigecycline, doxycycline, minocycline, or meropenem against clinical *A. baumannii* isolates [78,79]. Plant extracts provide a novel treatment option for tigecycline-resistant pathogens, but issues such as concentration dependence and therapeutic safety need to be addressed.

### 6.3. Phages

Phage is a class of viruses specifically infecting bacteria (Figure 5). With the rapid increase in antibiotic usage and antibiotic resistance, phage therapy is highly anticipated for treating MDR *Acinetobacter* infections [80,81]. Promisingly, a novel lytic phage, vABWU2101, demonstrated a good antibacterial activity against MDR *A. baumannii* and biofilms, especially with tigecycline [82]. Phage Abp1 was effective in lysing pan-drug resistant *A. baumannii* and showed no toxicity on HeLa and THP-1 cells [83]. In a mouse model of wound infection with *A. baumannii*, wound healing was accelerated when phage Abp1 was given locally [83]. In a systemic infection model, the survival rate of mice treated with phage Abp1 was 100%, and mice livers and kidneys were essentially free of bacteria [83]. Phage cocktail, which combines multiple phages into a cocktail, can overcome the narrow host range [84]. In Europe, a four-phage cocktail composed of Highwayman, Silvergun, Fanak, and PhT2-v2 was effective against the most prevalent ST2-KL3 *A. baumannii* lineage in *Galleria mellonella* and mouse models [84]. The survival rate of cocktail-treated mice with phage PBAB08, phage PBAB25, and other phages was increased by 2.3-fold in a mouse model of nasal infection with MDR *A. baumannii* [85].

Despite some achievements in phage therapy under experimental conditions, only one phage completed a clinical double-blind and randomized study of *A. baumannii* infections in Phase I/IIa registered on ClinicalTrials.gov (https://clinicaltrials.gov/, accessed on 23 September 2025). On one hand, the deficiencies of host range and standardization of quality evaluation and large-scale production challenge the clinical application of phage therapy [81]. For application risk and public safety, phage resistance has been discovered in recent years [86]. Meanwhile, the phage-mediated antibiotic resistance in *Acinetobacter* sp. bacteria cannot be ignored, especially colistin resistance and carbapenem resistance of global concern [87,88].

### 6.4. AMPs

Most AMPs have a composition of less than 100 amino acids and are non-specifically antibacterial by disrupting bacterial membrane [89,90]. A bacterial toxin CcdB-derived peptide, CP1-WT, showed high therapeutic efficacy in treating tigecycline-resistant *A. baumannii* infection [91]. OH-CATH30 and D-OH-CATH30 derived from King Cobra exhibited good antimicrobial activities against clinical *Acinetobacter* sp. isolates [92]. Tilapia piscidin 2 (TP2)-based AMPs, TP2-5 and TP2-6, significantly inhibited *A. baumannii* biofilms with low toxicity even after a consecutive passage [93]. A computational peptide omega76 was designed to combat tigecycline-resistant *A*. *baumannii* infection in mice without chronic toxicity [94]. Additionally, machine learning predicted nearly 1 million novel AMPs in the global microbiome, of which 79 out of 100 tested peptides were active in vitro [95]. Two days post-infection of *A. baumannii* in mice, lachnospirin-1, enterococcin-1, ampspherin-4, and reyranin-1 exhibited bactericidal activities close to that of polymyxin B [95]. There were 32 optimized AMPs also obtained from low-abundance human oral bacteria by deep learning, of which the most potent AMP pep-19-mod achieved over 95% reduction in bacterial loads in a murine thigh infection model [96]. Due to the limited production of natural AMPs, there is need for deep learning methods and synthetic technologies to modify peptide sequences for more potent AMPs [97,98].

### 6.5. CRISPR-Cas

CRISPR-Cas, consisting of repeats, spacers, and CRISPR-associated genes, has emerged as a promising tool for combating MDR pathogens (Figure 5). For instance, an I-Fb CRISPR-Cas system in *A. baumannii* was shown to degrade quorum sensing regulator mRNA, generating reactive oxygen species and reducing biofilm formation and efflux pump activity, which contributed to antibiotic susceptibility such as tetracyclines [99]. CRISPR-based tools are also developed for precise genome editing in *A. baumannii*, enabling scar-free mutagenesis in MDR strains [100,101]. A CRISPRi system was developed to knock down the essential genes in *A. baumannii* for bacterial control [102]. An integration of CRISPR-Cas gene editing with toxin-antitoxin module CreTA was designed to combat MDR *A. baumannii* [103]. However, challenges remain in delivery efficiency, particularly in complex clinical settings, and in avoiding off-target effects [104]. Notably, interdisciplinary collaboration is critical to address ethical concerns and streamline regulatory pathways for clinical adoption [105,106]. With continued innovation, CRISPR-based approaches may revolutionize the treatment of *Acinetobacter* infections, offering a scalable alternative to traditional antibiotics in the fight against tigecycline-resistant pathogens [107].

## 7. Conclusions

In summary, the *tet*(X) genes, especially *tet*(X3), have spread across multiple *Acinetobacter* species around the world, threatening the clinical application of tetracycline antibiotics, public health, and food safety. Besides plasmids, IS*CR2* is a key element involved in the horizontal transmission of *tet*(X) genes. There is an urgent need for rapid detection technologies to monitor the *tet*(X)-positive *Acinetobacter* sp. strains in different ecological niches, including human, animal, food, and environmental samples. By deep learning tools and screening small-molecule libraries, some novel antibiotics are developed to treat MDR *Acinetobacter* infections. Under the global policy background of reducing and limiting antibiotics, alternative non-antibiotic approaches such as plant extracts, phages, AMPs, and CRISPR-Cas technologies are promising in eliminating the threat of *tet*(X)-mediated tigecycline-resistant in *Acinetobacter* sp. pathogens.

## Data Availability

No new data were created or analyzed in this study. Data sharing is not applicable to this article.

## Abbreviations

The following abbreviations are used in this manuscript:

MDR	Multidrug-resistant
AMPs	Antimicrobial peptides
CRAb	Carbapenem-resistant *A. baumannii*
USFDA	United States Food and Drug Administration
MIC	Minimum inhibitory concentration
FAD	Flavin adenine dinucleotide
NADPH	Reduced nicotinamide adenine dinucleotide phosphate
WGS	Whole genome sequencing
IS	Insertion sequence
OTG	Orange to green
MALDI-TOF MS	Matrix-assisted laser desorption ionization–time of flight mass spectrometry
MCP	Macrocyclic peptide
TP2	Tilapia piscidin 2
